# Phenolic Contents and Antioxidant Properties of *Bauhinia rufescens*, *Ocimum basilicum* and *Salvadora persica*, Used as Medicinal Plants in Chad

**DOI:** 10.3390/molecules29194684

**Published:** 2024-10-02

**Authors:** Hissein Hassan Abdel-razakh, Gaymary George Bakari, Jin-Soo Park, Cheol-Ho Pan, Abubakar Shaaban Hoza

**Affiliations:** 1 Department of Veterinary Microbiology, Parasitology and Biotechnology, College of Veterinary Medicine and Biomedical Sciences, Sokoine University of Agriculture, Chuo Kikuu, Morogoro P.O. Box 3019, Tanzania; razakh10@gmail.com (H.H.A.-r.); abshoza@gmail.com (A.S.H.); 2 SACIDS Foundation for One Health, Sokoine University of Agriculture, Chuo Kikuu, Morogoro P.O. Box 3015, Tanzania; gaymary.bakari@sua.ac.tz; 3 Natural Product Informatics Research Center, KIST Gangneung Institute of Natural Products, Gangneung 25451, Republic of Korea; panc@kist.re.kr; 4 Department of Veterinary Physiology, Biochemistry and Pharmacology, College of Veterinary and Biomedical Sciences, Sokoine University of Agriculture, Morogoro P.O. Box 3017, Tanzania; 5 Natural Product Applied Science, KIST School, University of Science and Technology, Gangneung 25451, Republic of Korea

**Keywords:** antioxidant activity, *Bauhinia rufescens*, *Ocimum basilicum*, *Salvadora persica*, total flavonoid contents, total phenolic contents, total tannin contents

## Abstract

The plants *Bauhinia rufescens*, *Ocimum basilicum* and *Salvadora persica* are well known in traditional African medicine, and particularly in traditional Chadian medicine. They are commonly used to treat infectious diseases, inflammatory diseases, fevers, gastroenteritis and other medical conditions. The aim of this study was to perform a phytochemical screening to determine the antioxidant properties of different extracts and fractions from the three plants. Ethanolic extracts and solvent fractions were prepared and analyzed for total phenolic content (TPC), total flavonoid content (TFC) and total tannin content (TTC). LC-MS and an online screening HPLC-ABTS system identified phytochemicals with antioxidant activities. DPPH and ABTS reduction methods were used to test the extracts and fractions for their antioxidant potential. The results showed that the TPC of *O. basilicum* was higher than that of *B. rufescens*, ranging from 64.70 ± 5.2 to 411.16 ± 8.11 mgGAE/g DW. *B. rufescens* extracts and fractions, on the other hand, showed higher TFC, ranging from 69.5 ± 5.3 to 408.26 ± 8.42 mgQE/g DW, and higher TTC, ranging from 4.57 ± 2.45 to 62.19 ± 4.7 mgTAE/g DW. The maximum TPC, TFC and TTC in both plants were recorded in the ethyl acetate fractions. *S. persica* extracts and fractions showed a very low quantity of TPC, TFC and TTC. Based on LC-MS and HPLC-ABTS analysis, rosmarinic acid was identified as the major component in the extracts and all fractions of *O. basilicum,* and epicatechin, procyanidin B and quercetin were found in *B. rufescens*. *S. persica* did not exhibit specific substances with antioxidant activity and was therefore not considered for further assays. DPPH and ABTS results showed that ethyl acetate fractions of *B. rufescens* and *O. basilicum* have the strongest antioxidant activities. This study indicates that *B. rufescens* and *O. basilicum* are good sources of phytochemicals with antioxidant properties, suitable for medicinal use in Chadian communities. Additionally, the antioxidant-rich extracts from these plants hold significant potential for cosmetic development, enhancing skin health and protecting against oxidative-stress-induced damage.

## 1. Introduction

Medicinal plants have played an important role in maintaining public health and in the survival of humanity since ancient times [1]. Across all continents, medicinal plants have been used as remedies for a wide range of human diseases for centuries [2]. In Africa, over 90% of the population currently rely on plant-based medications for their primary healthcare needs as a result of the high cost of modern medicines, and also due to the strong healing powers that they possess, along with a lack of side effects [3]. The healing power of the plants is attributed to the chemical compounds they contain, with known active principles such as alkaloids, phenolic compounds, essential oils, vitamins, quinnones, saponins, tannins, etc. Phenolic compounds are particularly used as antioxidants in the pharmaceutical, cosmetic and food fields for their beneficial effects on health [4]. In addition, phenolic compounds also possess various other biological properties, such as antimicrobial, anti-inflammatory, antioxidant, antiviral, anti-cancer and hepato-protective properties [4,5].

**Figure 1:**
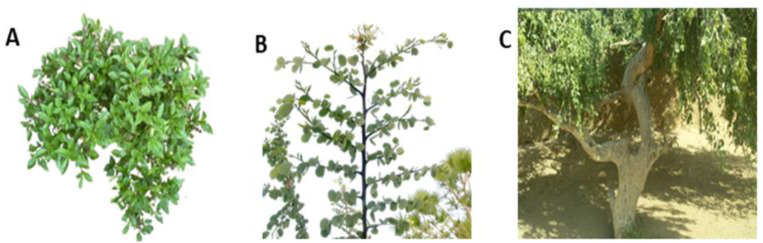
Three medicinal plants commonly used in Chad that were examined in this study: (**A**) *O. basilicum* plant, (**B**) *B. rufesens* plant and (**C**) *S. persica* shrub.

*Bauhinia rufescens* (Figure 1A), also known as ‘koulkoul’ in Chadian Arabic, is a highly branching bush from the Fabaceae family. The plant’s bark is pale gray and often scaly. The persistent and biloba leaves alternate. The inflorescences in corymbe are around 5 cm long. The fruit is a dark brown spiral shape with four to ten seeds. The plant’s root system pivots, and deep roots provide access to the pad. This plant is a species of arid and semi-arid environments, particularly the Sahel and Sahel–Sudan areas, found generally in the countries of West Africa, Central Africa and the Maghreb, and more precisely in Niger, Burkina Faso, Algeria, Mauritania, Guinea, Sierra Leone, Mali, Côte d’Ivoire, Ghana, Benin, Nigeria, Cameroon, Chad, Sudan and Ethiopia, on various dry soils, generally sandy, lateral or clay [6]. Traditional medicine uses macerated leaves for stomachic, anti-diarrheal, febrifuge and anti-dysenteric effects. They are also used in decoctions to treat eye problems and hypertension. A decoction of *B. rufescens* leaves has also been reported to cure diabetes, mycoses and fibrosis [5]. The boiled and chopped roots are used as a drink to treat leprosy, syphilis and other venereal infections [6]. The bark is used to treat smallpox and other chest illnesses. Chadians utilize *B. rufescens* to cure typhoid fever, malaria, diabetes and other common illnesses [6].

*Ocimum basilicum* (Figure 1B), also known as ‘am-rihanna’ in Chadian Arabic, is an annual aromatic plant that originated in India and South Asia and has been grown for over three thousand years. It is a herbaceous plant that is woody and very branched, reaching heights of up to one meter. The opposite leaves are denticulated, while the pectorals are oblong or obovate, spotted at the base and acuminated at the top. The inflorescence in terminal spiciform racem is loose and composed of small groups of whitish flowers with two lips: the upper part is more developed and has four teeth on top, while the lower part is short and rounded. The whole plant and its essential oil have several traditional medical uses, particularly in Africa and India. A decoction of the leaves is used to treat a variety of diseases, including warts, worms, bronchitis, headaches, coughs, sunburns, diarrhea and chronic dysentery [6].

*Salvadora persica* (Figure 1C), known locally as ‘Miswak’, is a small tree or shrub in the Salvadoraceae family with opposite leaves and inflorescences in long, more or less branched clusters; tetrameter flowers, cupuliform, with yellowish-green short petals and altering staminodes in the shape of short teeth; endocarpic, crustaceous and single-seeded oval drupe. *S. persica* is a native Middle Eastern plant. This tree can be found throughout Africa and Asia, including in Senegal and Mauritania, Lake Chad, Nigeria, Mozambique, South Africa, India, Pakistan, Saudi Arabia and Iran. In Chad, the roots are used to clean teeth, while the leaves’ infusion or decoction is used to treat diarrhea and gastrointestinal ailments. Boils can be cured locally by combining powdered root and water. In Algeria, Egypt and Libya, syphilis has been treated with a 40-day regimen of powdered leaves, millet flour and honey, which was consumed in small scoops every morning. In Tanzania, oral candidiasis is treated locally three times a day with a paste made from powdered bark powder and table oil [6].

The therapeutic value of these plants is related to their secondary metabolites, especially phenolic compounds, whose concentration of such molecules may differ from one plant to another and from one part of the same plant to another.

This research aimed to determine the total phenolic, total flavonoid and total tannin contents, to perform LC-MS and HPLC-ABTS and finally to evaluate the antioxidant properties of *B. rufescens*, *O. basilicum* and *S. persica*.

## 2. Results

### 2.1. Yields of Extracts

The various extracts and fractions obtained after evaporation were weighed to determine their final dry weights. The yield was determined for 300 g of plant material. By considering “M” to be the mass of powder used for extraction and m the mass of extract obtained after extraction and fractionation, the extraction yield (%) can then be estimated using the following formula:

Yield (%) = (m/M) × 100

The results are shown in Table 1 and indicate that the aqueous fractions of all the three plants tested showed relatively high extraction yields compared to any other extracts or fractions.

### 2.2. Total Phenolic, Total Flavonoid and Total Tannin Contents

The total phenolic, total flavonoid and total tannin contents were determined using the Folin–Ciocalteu and aluminum trichloride (AlCl_3_) methods, as appropriate. The quantities of total phenols, total flavonoids and total tannins were determined on the basis of linear regression equations for each calibration curve, expressed in milligrams equivalent of gallic acid (GAE), milligrams equivalent of quercetin (QE) and milligrams equivalent of tannic acid (TAE), respectively.

**Figure 2:**
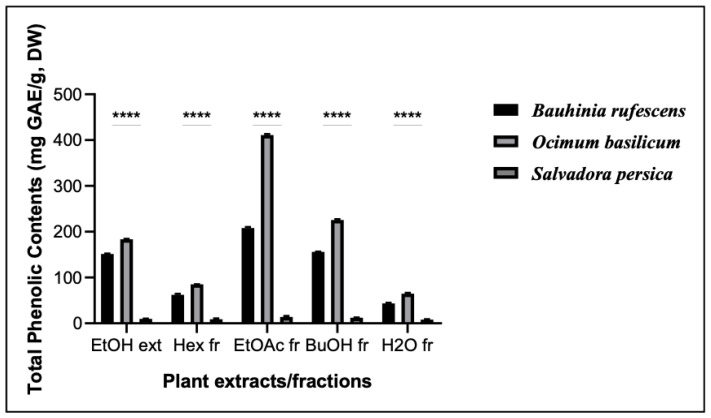
Total phenolic content of extracts and fractions of *B. rufescens, O. basilicum* and *S. persica*. EtOH ext: ethanolic extracts; Hex fr: hexane fractions; EtOAc fr: ethyl acetate fractions; BuOH fr: butanol fractions; H_2_O Fr: aqueous fractions. **** represent statistically different results among extracts or fractions of the three plants (*p* < 0.05).

**Figure 3:**
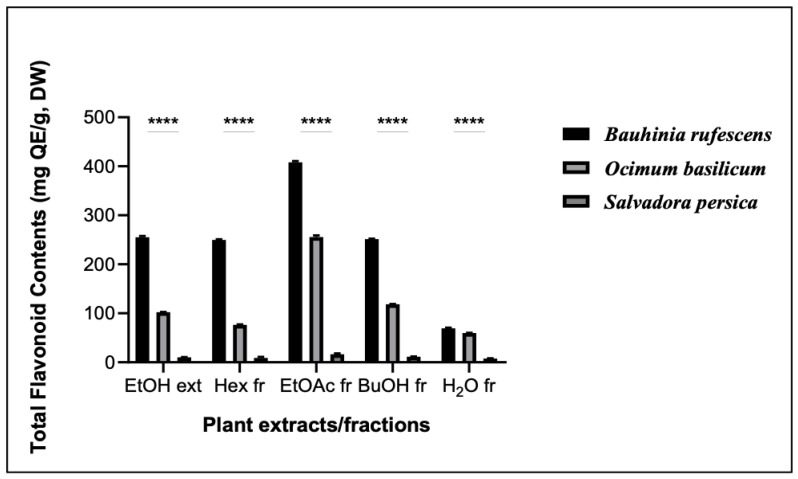
Total flavonoid contents of extracts and fractions of *B. rufescens, O. basilicum* and *S. persica*. EtOH ext: ethanolic extracts; Hex fr: hexane fractions; EtOAc fr: ethyl acetate fractions; BuOH fr: butanol fractions; H_2_O Fr: aqueous fractions. **** represent statistically different results among extracts or fractions of the three plants (*p* < 0.05).

**Figure 4:**
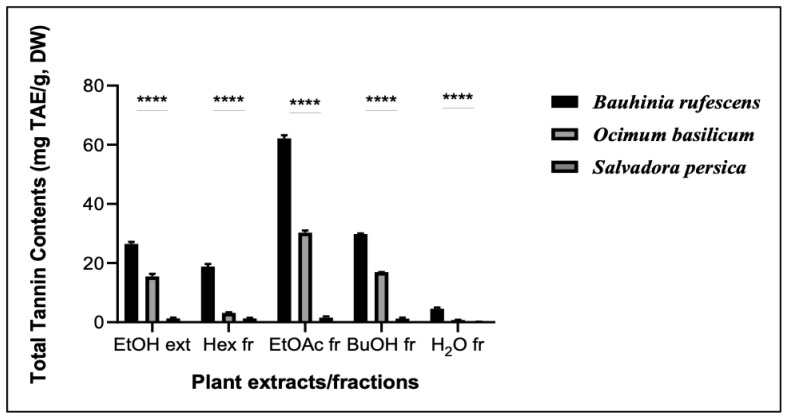
Total tannin contents of extracts and fractions of *B. rufescens, O. basilicum* and *S. persica*. EtOH ext: ethanolic extracts; Hex fr: hexane fractions; EtOAc fr: ethyl acetate fractions; BuOH fr: butanol fractions; H_2_O Fr: aqueous fractions. **** represent statistically different results among extracts or fractions of the three plants (*p* < 0.05).

The results of these assays are shown in Figure 2, Figure 3 and Figure 4. The TPC of *O. basilicum* was higher than that of *B. rufescens*, ranging from 64.70 ± 5.2 to 411.16 ± 8.11 mgGAE/g DW. However, *B. rufescens* extracts and fractions showed higher flavonoid content, ranging from 69.5 ± 5.3 to 408.26 ± 8.42 mgQE/g DW, and higher tannin content, ranging from 4.57 ± 2.45 to 62.19 ± 4.7 mgTAE/g DW. Maximum phenolic contents in both plants were recorded in the ethyl acetate fractions, and minimum contents in the aqueous fractions. A similar trend was observed for flavonoid and tannin contents, ethyl acetate being the best solvent for phenolic, flavonoid and tannin extractions. *S. persica* extracts and fractions contain only trace amounts of TPC, TFC and TTC.

### 2.3. LC-MS/HPLC-ABTS

In order to screen the three plants for bioactive compounds with antioxidant potential, extracts and fractions were analyzed by LC-MS and on-line HPLC-ABTS.

**Figure 5:**
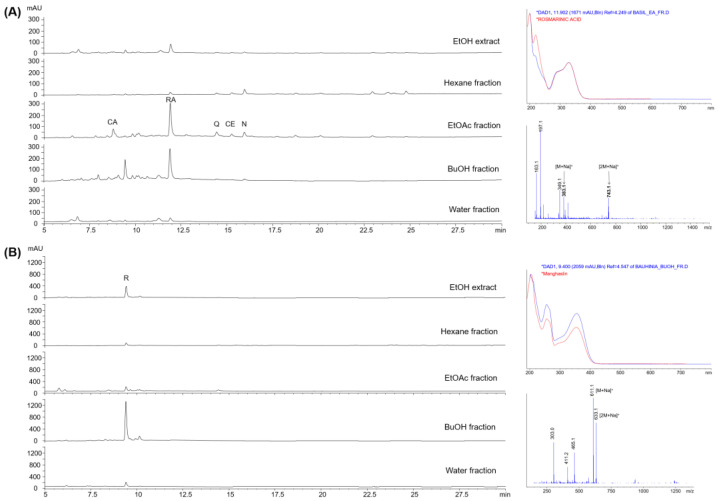
LC-MS data of the extract and fraction of *O. basilicum* (**A**) and *B. rufescens* (**B**) (**A**) HPLC chromatograms measured at 254 nm of EtOH extract and solvent fractions of *O. basilicum*, UV–vis absorption spectra of a compound with a retention time of 11.9 min of *O. basilicum* and rosmarinic acid from in-house UV library and mass spectrum of the compound with retention time of 11.9 min, indicating molecular mass of 360 daltons, identical to rosmarinic acid. CA: caffeic acid; RA: rosmarinic acid; Q: quecetin; CE: caffeic acid ethyl ester; N: neopetoidin B. (**B**) HPLC chromatograms at 254 nm of EtOH extract and solvent fractions of *B. rufescens*, UV–vis absorption spectra of a compound with retention time of 9.4 min of *B. rufescens* and manghaslin from in-house UV library and mass spectrum of the compound with retention time of 9.4 min, indicating molecular mass of 610 daltons, identical to rutin. R: rutin.

In *O. basilicum*, rosmarinic acid was identified as the major component in the extracts and all fractions based on comparisons with our in-house UV library and mass spectral analysis (Figure 5A). In addition, caffeic acid (Rt. 8.8 min), quercetin (Rt. 14.4 min), caffeic acid ethyl ester (Rt. 15.3 min), neopetoidin B (Rt. 16.0 min) and polymethoxyflavones (Rt. 19.1, 20.4 min) such as cirsilineol and eupatorin with a molecular weight of 344 daltons were identified in the EtOAc fractions. Further isolation from ethyl acetate fraction using preparative HPLC proved the presence of rosmarinic acid and neopetoidin B through ^1^H NMR and high-resolution mass spectrometric data (Figures S1 and S2).

The HPLC chromatogram at 254 nm showed a simple composition of *B. rufescens* (Figure 5B). A substance with a molecular weight of 610 at 9.4 min was identified as the main component, and its UV–Vis absorption spectrum was very similar to that of manghaslin, a type of flavonoid glycoside. The substance was identified as rutin, a flavonoid glycoside, by preparative HPLC based on ^1^H NMR and high-resolution MS analysis (Figure S3).

A variety of substances ranging in molecular weight from 195 to 478 were detected in the extracts and fractions of *S. persica* (Figure S4). Of them, the substances with a retention time of 14.5 min and 21.5 min were likely to be persicaline and N-benzyl O-ethyl thiocarbamate isolated from *S. persica* [7]. But further chemical identification was not continued because subsequent HPLC-ABTS results did not reveal any antioxidants.

**Figure 6:**
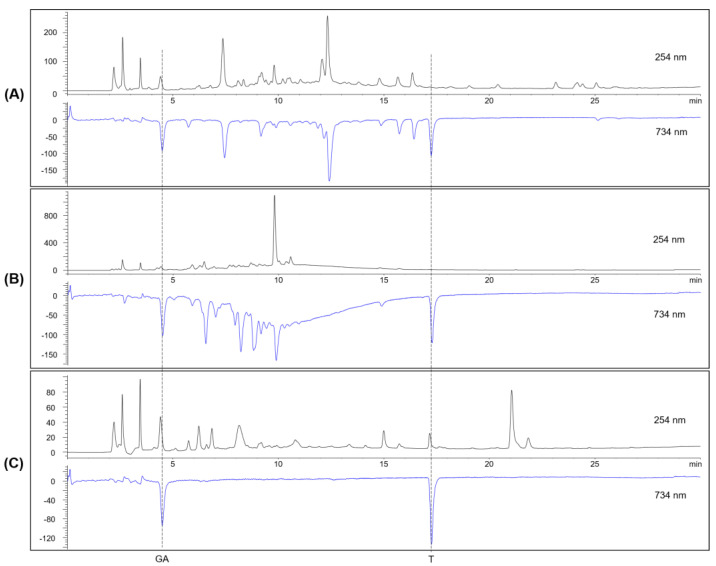
Radical-scavenging activities of the ethanolic extracts of (**A**) *O. basilicum,* (**B**) *B. rufescens* and (**C**) *S. persica* assessed through an online ABTS assay. The compounds corresponding to the two dashed lines are gallic acid (GA) and trolox (T) as internal standards.

With the online HPLC-ABTS system, the antioxidant activity scavenging ABTS radical detected as a negative peak at 734 nm was identified in the ethanolic extracts of *O. basilicum* and *B. rufescens* (Figure 6A,B, Figures S6 and S7). For *O. basilicum*, the peaks corresponding to caffeic acid, rosmarinic acid, caffeic acid ethyl ester and neopetoidin B found in the LC-MS analysis above demonstrated excellent radical-scavenging activity. In the case of *B. rufescens*, as expected, the main component, rutin, showed strong antioxidant activity, but there were various antioxidants that were not detected at 254 nm. Further examination of the LC-MS data of *B. rufescens* identified substances with molecular weights of 290, 578 and 302 which were predicted to be epicatechin, an epicatechin dimer (procyanidin B) and quercetin, respectively. The presence of epicatechin was confirmed by ^1^H NMR and high-resolution MS (Figure S5).

The results from *S. persica* did not show any peaks corresponding to significant antioxidants other than gallic acid and trolox, which were added as internal standards at a wavelength of 734 nm (Figure 6C and Figure S8). Therefore, the plant was excluded from further antioxidant-related experiments.

### 2.4. DPPH and ABTS Radical Reduction

The DPPH and ABTS radical-scavenging activity of all the extracts and fractions were further assessed by measuring the reduction of the DPPH radical to DPPH-H and of ABTS to the ABTS+ compounds. All the extracts and fractions showed antioxidant activity (Table 2), with ethyl acetate fractions from both plants showing lower IC_50_. It should be emphasized that the lower the IC_50_ value, the more powerful the extract or fraction is considered to be as an antioxidant.

## 3. Discussion

There is steadily growing interest in the use of bioactive compounds derived from natural sources as therapeutic agents to promote public health and treat various diseases. Antioxidant phytochemicals are of particular interest in the development of pharmaceuticals and cosmeceuticals. The main objective of this study was to determine the total phenolic, total flavonoid and total tannin contents, to screen the chemical contents and to assess the antioxidant properties of extracts and fractions of *B. rufescens*, *O. basilicum* and *S. persica*. The choice of these plants as study materials was guided by their common use in traditional medicine in Chad for the treatment of various diseases. In Africa, medicinal plants are still often used as medicines, so there is a need to better identify and understand the components responsible for their effects.

The yields obtained in the present study (Table 1) indicate that the aqueous fractions from all the three plants gave higher yields; respectively, *B. rufescens* aqueous fractions gave 13.3%, *O. basilicum* aqueous fractions yielded 10.44% and *S. persica* aqueous fractions produced 9.27%. The variations in yield of extracts and/or fractions are attributable to a wide range of different parameters, including the plant species, the plant organ used for extraction and fractionation, the drying temperature and other conditions, the extraction methods used, the choice of extraction or fractionation solvent and the metabolite content of each species. Among these fractions, the extraction yield of *S. persica* ethyl acetate fractions was the lowest (0.15%). These results are in agreement with the results obtained by Mahamat et al., with acetonic extracts of *B. rufescens* [8]; by Coelho et al., with methanolic extracts of *O. basilicum* [9]; and by Aissaoui and Maamri, with extracts of *S. persica* [10].

Polyphenols, including flavonoids and tannins, constitute a broad class of secondary metabolites with a variety of different structures. They are synthesized in the shikimic acid and pentose phosphate of plants through the metabolization of phenylpropanoids [11,12]. They contain benzene rings with one or more hydroxyl substituents, and range from simple phenolic molecules to highly polymerized compounds [12]. They are also involved in food digestibility and in the physiological use of proteins with which tannins combine. The ability of a plant species to resist attack by insects and micro-organisms is often correlated with its phenolic compound content [13]. Polyphenolic compounds are increasingly used in therapeutics due to the fact that they possess antioxidant, anti-inflammatory, anticarcinogenic, antiatherogenic, antiviral, analgesic, antibacterial, vasodilatory and anti-allergenic potentials.

In this study, total phenolic content, total flavonoid content and total tannin content were determined. The results have shown that all the extracts and fractions from *B. rufescens* and *O. basilicum* contain phenolic, flavonoid and tannin content, with ethyl acetate fractions of *O. basilicum* leaves possessing the highest quantity of total phenolic content and *B. rufescens* ethyl acetate fractions showing the highest flavonoid and tannin contents. The presence of phenolic, flavonoid and tannin contents in *B. rufescens* and *O. basilicum* extracts and fractions has also been reported by previous studies [6,8,14] but their antioxidant molecules have rarely been accurately characterized. *S. persica* extracts and fractions showed only very slight amounts of total phenols, total flavonoids and total tannins.

Extracts and fractions were analyzed with LC-MS and online HPLC-ABTS to search for bioactive compounds in the three plants. These two combined methods enabled a rapid identification of the presence of specific compounds with antioxidant potential in a given mixture through post-column mixing of the individual analytes with radical solutions. After using these methods, it was easy to detect the active components present in each extract or fraction. The intense peaks obtained from the LC-MS analysis indicated the presence of rosmarinic acid containing caffeic acid in both extracts and fractions. Caffeic acid is a phenolic acid widely recognized for its numerous health benefits. The presence of rosmarinic acid, caffeic acid and polymethoxyflavones as the predominant secondary metabolites in *O. basilicum* has also been mentioned in previous studies [15,16,17,18,19]. The LC-MS results, however, have shown that the major compound in *B. rufescens* ethyl acetate fractions is rutin, a flavonoid glycoside. Flavonoids in general are recognized as one of the most important phytochemical constituents among the other secondary metabolites of species within the genus [20,21,22]. *S. persica* extracts and fractions, for their part, did not contain any detectable substances.

The presence of chemical compounds with antioxidant potential, detected by a negative peak at 734 nm in the HPLC–ABTS system, was noted in the ethyl acetate fractions of *B. rufescens* and *O. basilicum*. By means of activity-guided chromatographic separation and isolation from the plant extracts and fractions, the online HPLC-ABTS assay has proved to be a rapid and effective method for identifying active compounds with antioxidant potential. *S. persica* extracts and fractions, however, did not contain any detectable substances with potential antioxidant activity; hence, they were excluded from further antioxidant experiments.

The results obtained in this study with the DPPH and ABTS methods showed that all extracts and fractions have antioxidant activity, which varies from one plant to another and, for the same plant, from one extract or fraction to another. As shown in Table 2, ethyl acetate fractions from *B. rufescens* and *O. basilicum* demonstrated the highest free-radical-neutralizing effect. These findings confirm the results obtained with TPC, TFC, TTC, LC-MS and HPLC-ABTS screening, with ethyl acetate giving the most active fractions. Ethyl acetate was found to be an excellent solvent for the extraction of phenolic compounds, flavonoids and tannin, which were, for their part, responsible for the antioxidant potential in these plants. This observation is corroborated by findings in the literature that ethyl acetate is the most suitable solvent for the extraction of plant polyphenols [23,24].

Comparing the DPPH and ABTS methods, the antioxidant capacity of each plant extract and fraction determined by the ABTS assay was found to be significantly higher than the values obtained with the DPPH method. These results could be ascribed to the higher level of detectability, repeatability and sensitivity of the ABTS test in the determination of the antioxidant potential of extracts and fractions in comparison with the DPPH test [25].

## 4. Materials and Methods

### 4.1. Extraction and Fractionation

#### 4.1.1. Extraction

*B. rufescens* and *O. basilicum* leaves and *S. persica* roots were obtained from the N’Djari area in N’Djamena, Republic of Chad. They were authenticated at the department of plant biology at the university of N’Djamena. The leaves and roots were washed under running tap water, cut into small pieces, ground into powder and placed in contact with aqueous ethanol (70% *v*/*v*). The mixture was sonicated for 30 min, then incubated at room temperature for 24 h, and then filtered using Whatman Filter n°4 (Whatman, Maidstone, UK). The extraction was repeated twice, and the mixtures were combined and evaporated using a rotary evaporator. The final ethanolic extracts were obtained, from which 10% were freeze-dried and kept in the refrigerator for further uses and the remaining 90% were used for fractionation.

#### 4.1.2. Fractionation

Distilled water was added to the 90% of extract of each plant in a flask to adjust it to 500 mL, and then 1500 mL of hexane was added to that. The mixture was shaken and incubated for 3 h at room temperature, and then, the hexane layer was collected, evaporated and stored for the different assays.

Next, 1500 mL of ethyl acetate was added to the remaining water layer. The mixture was incubated for 3 h at room temperature, and the ethyl acetate layer was collected for evaporation. The ethyl acetate fraction was obtained and stored.

Then, 1500 mL of concentrated butanol was added to the remaining water layer. The mixture was incubated for 3 h at room temperature, and then the butanol layer was collected for evaporation. The butanol fraction was obtained and stored. The remaining aqueous layer was also evaporated to obtain the water fraction.

**Figure 7:**
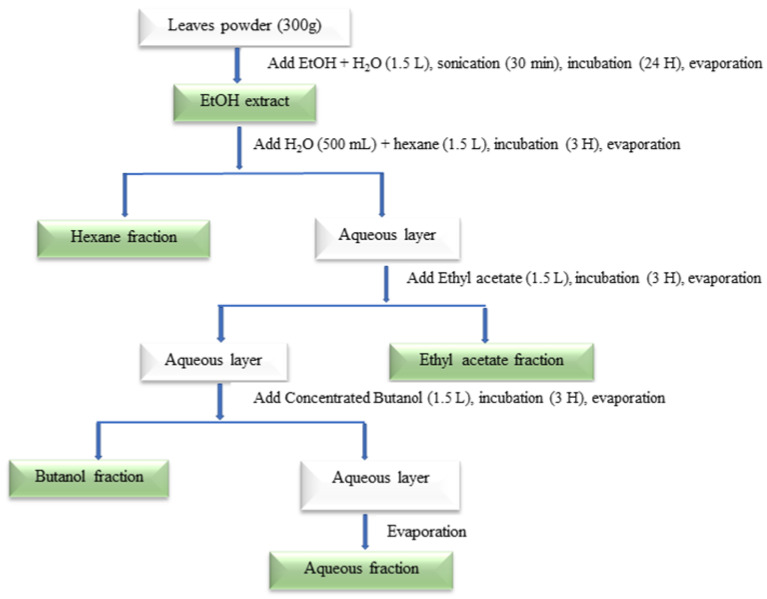
Schematic representation of the extraction and fractionation of plants.

The following figure (Figure 7) shows the different stages of the process for obtaining extracts and fractions.

### 4.2. Total Phenolic Content Determination

The phenolic contents of extracts and fractions were assessed using the Folin–Ciocalteu method as described by Phuyal et al. [26], with suitable modifications. The plant extracts and fractions were dissolved in DMSO to obtain a stock solution of 5 mg/mL.

From the stock solution, a total of 10 µL was added to 10 µL of 1N Folin in a 96-well plate. The mixture was then kept in the darkroom for 5 min. The mixture was allowed to incubate for 1 to 2 h after mixing with 80 µL of 7.5% sodium carbonate (NaHCO_3_). At 760 nm, the absorbance value was measured spectrophotometrically (SpectraMax ABSPlus, San Jose, CA, USA) and the phenolic content was assessed in triplicate. From different concentrations (12.5 to 800 µg/mL) of gallic acid (standard solution), a standard curve was obtained, y = 0.0048x + 0.0244, (R^2^ = 0.9998), where y represents the absorbance detected at 760 nm and x represents the total phenolic content, which was expressed as the percentage of total gallic acid equivalents per gram of extract (mg GAE/g).

### 4.3. Total Flavonoid Content Determination

With minor modifications, the total flavonoid content was determined using the colorimetric aluminum chloride (AlCl_3_) assay method described previously [27]. Briefly, on a microplate with 96 wells, to 10 μL of sample extracts or fractions (5 mg/mL) was added 10 μL of sodium nitrite (NaNO_2_, 5%), which was permitted to react for 6 min at ambient temperature and in the dark. Then, the mixture was allowed to react with 20 μL of aluminum chloride (AlCl_3_·6H_2_O, 10%) for 5 min, prior to adding 50 μL of sodium hydroxide (NaOH, 1M) and 110 μL of distilled water. The TFC was determined by measuring the absorbance at 510 nm with a multi-detection microplate reader (SpectraMax ABSPlus) after 10 min of incubation. Quercetin (12.5 to 800 µg/mL) was used as a standard in this experiment to develop the calibration curve as follows: y = 0.0005x − 0.0008 (R2 = 0.9996), where y represents the absorbance detected at 510 nm and x represents the flavonoid content. Total flavonoids were expressed as milligram Quercetin equivalent (QE) per gram dry weight (DW) of the extract using the dilution factor [27].

### 4.4. Total Tannin Content Determination

The total tannin content of plant extracts and fractions was quantified through the Folin–Denis method, using tannic acid as a standard. The sample and standard absorbance values were recorded at 725 nm using a spectrophotometer. The total tannin content of the plant extracts/fractions is expressed as mg Tannic Acid Equivalent (TAE)/dry extract.

### 4.5. Liquid Chromatography and Mass Spectrometry (LC-MS) Analysis of Extracts and Fractions and the NMR Experiment for Isolated Compounds

HPLC was carried out using the Agilent 1200 system LC-MS quadrupole 6120 (USA) with a Phenomenex Luna C18(2) 5 µm (4.6 × 150 mm) column. The mobile phase was composed of solvent A, acetonitrile, and solvent B, water. The flow rate was 0.7 mL. Elution was performed from 10% A and then increased to 100% A after 30 min. The injection volume was 10 µL for positive mode measurements. Major compounds were isolated using preparative HPLC with a Luna C18(2) 10 µm (10 × 250 mm) column, and NMR spectra were recorded using 500 MHz Bruker Avance NEO spectrometer (Bruker Biospin, Ettlingen, Germany). High-resolution mass spectrometric data were obtained using a Q Exactive Hybrid Quadrupole-Orbitrap Mass Spectrometer (Thermo Scientific, Dreieich, Germany).

### 4.6. Online High-Performance Liquid Chromatography (HPLC)–ABTS+ Based Assay

In order to assess the antioxidant activity of the extracts and fractions directly, the online HPLC-ABTS screening system was used. The system is composed of an Agilent 1200 analytical HPLC system (Agilent Technologies, Santa Clara, CA, USA) equipped with an additional pump to provide the ABTS radical solution. A stock solution of 2 mM ABTS containing 3.5 mM potassium persulfate was prepared in water and diluted 8-fold in HPLC-grade water for the ABTS radical reagent. To stabilize the radicals, this solution was incubated overnight in the dark at room temperature. The antioxidant capacity was measured according to a previous method [22]. In brief, 10 μL of extracts or fractions of the three plants were injected into the online HPLC-ABTS system. Individual compounds were separated with a column (Phenomenex Luna C18(2) 5 µm (4.6 × 150 mm)) in a solvent system of 0.1% TFA in acetonitrile (A) and 0.1% TFA in water (B) with a flow rate of 1 mL/min. The gradient conditions were as follows: 0–10 min, 10% A; 10–30 min, 100% A; and then return to initial conditions. The column was maintained at 25 °C for the duration of the sequence. The ABTS radical solution was supplied at a flow rate of 0.7 mL/min. The chromatogram was recorded as the positive peak, and the visible detector was set at 734 nm to measure the decrease in ABTS radicals as the negative peak. The data were analyzed using ChemStation software B.04.03 (Agilent Technology, Santa Clara, CA, USA).

### 4.7. The 2,2-diphenyl-1-picrylhydrazyl (DPPH) Radical Reduction Test

The free radical-scavenging capacity was determined colorimetrically using the DPPH decolorization method, as described previously but with minor modifications [28]. In a 96-microwell plate, 90 µL of a freshly prepared methanolic solution of DPPH (0.1 mM) was mixed with 10 µL of extracted samples prepared at concentrations ranging from 50 µg/mL to 1000 µg/mL. After incubation for 10 min at room temperature in the dark, the absorbance of the reagent solution was measured using a multi-detection microplate reader (SpectraMax ABSPlus) set at 515 nm. To calculate the percentage (%) inhibition of the DPPH radical, the following equation was used:

%inhibition = (1 − (AS/AC)) × 100

(AC is the absorbance of the control (methanol and DPPH reagent) and AS is the absorbance of the tested extract sample).

DPPH IC_50_ values (sample concentration required to inhibit 50% of DPPH radicals) were obtained by regression analysis extrapolation. Antioxidant evaluation was based on this IC_50_ value.

### 4.8. Determination of the 2,2′-azino-bis (3-ethylbenzothiazoline-6-Sulfonic Acid) (ABTS) Antioxidant Activity

To prepare the antioxidant reagent, we reacted 14 mM of 2,2-azino-bis (3-ethylbenzothiazoline-6- sulfonate) (ABTS) dissolved in water with a 4.9 mM potassium persulfate solution. The mixture was then incubated overnight at 4 °C in the dark to generate free radicals. The antioxidant test was conducted as described in a previous protocol, with minor modifications [22]. To briefly summarize, 10 µL of extracted samples prepared at concentrations ranging from 50 µg/mL to 1000 µg/mL in a 96-micro-well plate were reacted with 90 µL of ABTS+ reagent. Plates were incubated in the dark for 10 min before absorbance was measured at 734 nm using a multiple detection microplate reader (SpectraMax ABSPlus). The percentage (%) radical-scavenging activity of DW extracts or fractions was calculated as described below:

% inhibition = (1 − (AS/AC)) × 100

(AC is the absorbance of the control, a mixture of ethanol and ABTS reagent, and AS is the absorbance of the tested extract sample).

The ABTS IC_50_ values (sample concentration required to inhibit 50% of ABTS radicals) were obtained by extrapolating from a regression analysis. Antioxidant activity was assessed on the basis of this IC_50_ value.

### 4.9. Statistical Analysis

All the results are expressed as mean ± standard deviation (SD). The comparison of the groups was carried out using Prism 9 software. Values are considered to be significantly different at a level of less than 5% (*p* < 0.05).

## 5. Conclusions

*B. rufescens*, *O. basilicum* and *S. persica* are shrubby and herbaceous plants well known to the populations of several African countries, and Chad in particular. Renowned for their antimicrobial and anti-inflammatory activities, their aerial parts and roots are mainly used by indigenous populations to treat digestive disorders, wounds, dermatitis, oral hygiene, tooth decay, mycosis and other microbial infections and inflammations. These folk usages have drawn the diligent attention of scientists, who have conducted a number of studies in various different research laboratories to support the plants’ traditional usages and identify the active compounds associated with the plants’ properties. The in vitro tests in this study on TPC, TFC, TTC and the antioxidant potential of *B. rufescens*, *O. basilicum* and *S. persica* extracts and fractions have proved that *B. rufescens* and *O. basilicum* have important amounts of TPC, TFC and TTC, with higher results observed in the ethyl acetate fractions of the both plants, and strong antioxidant potential due to their specific phenolic compounds, supporting the practices of traditional Chadian medicine.

## Figures and Tables

**Table 1:** Yields from the three plant extracts and fractions (%).

Plant	Ethanol Extracts	Hexane Fractions	Ethyl Acetate Fractions	Butanol Fractions	Water Fractions
*B. rufescens*	2.01	0.97	1.14	5.27	13.3
*O. basilicum*	1.80	3.94	1.33	1.5	10.44
*S. persica*	1.33	0.24	0.15	0.68	9.27

**Table 2:** Radical-scavenging activity IC_50_ (µg/mL) of plant extracts and fractions.

Plant Extracts/Fractions		**DPPH**	**ABTS**
*B. rufescens*	EtOH ext	283.84 ± 0.00	210.55 ± 0.06
	Hex fr	483.18 ± 0.03	307.08 ± 0.05
	EtOAc fr	192.24 ± 0.07	119.93 ± 0.1
	BuOH fr	238.59 ± 0.07	202.6 ± 0.07
	H_2_O fr	718.49 ± 0.05	478.28 ± 0.05
*O. basilicum*	EtOH ext	294.68 ± 0.002	207.95 ± 0.001
	Hex fr	465.95 ± 0.002	318.72 ± 0.002
	EtOAc fr	199.51 ± 0.001	121.08 ± 0.002
	BuOH fr	278.14 ± 0.003	198.85 ± 0.01
	H_2_O fr	767.46 ± 0.005	470.90 ± 0.01
Gallic acid		2.7 ± 0.1	1.9 ± 0.5

DPPH: 2,2-Diphenyl-1-picrylhydrazyl; ABTS: 2,2′-azino-bis (3-ethylbenzothiazoline-6-sulfonic acid; EtOH ext: ethanolic extracts; Hex fr: hexane fractions; EtOAc fr: ethyl acetate fractions; BuOH fr: butanol fractions; H_2_O Fr: aqueous fractions. The values represent the average of three replicates ± SD.

## Data Availability

The data are available from the corresponding authors.

## Supplementary Materials

The following supporting information can be downloaded at: https://www.mdpi.com/article/10.3390/molecules29194684/s1, Figure S1. ^1^H NMR spectrum and high-resolution mass spectrum of rosmarinic acid isolated from *O. bacilicum*, Figure S2. ^1^H NMR spectrum and high-resolution mass spectrum of neopetoidin B isolated from *O. bacilicum*, Figure S3. ^1^H NMR spectrum and high-resolution mass spectrum of rutin isolated from *B. rufescens*, Figure S4. LC-MS data of extract and solvent fractions derived from *S. persica*. The molecular ions of mass spectrum corresponding to each peak are shown below the dotted line. Figure S5. ^1^H NMR spectrum and high-resolution mass spectrum of epicatechin isolated from *B. rufescens*, Figure S6. Chromatograms (254 nm) and radical scavenging activities (734 nm) of the ethanolic extracts and fractions of *O. basilicum* from online HPLC-ABTS system., Figure S7. Chromatograms (254 nm) and radical scavenging activities (734 nm) of the ethanolic extracts and fractions of *B. rufescens* from online HPLC-ABTS system., and Figure S8. Chromatograms (254 nm) and radical scavenging activities (734 nm) of the ethanolic extracts and fractions of *S. persica* from online HPLC-ABTS system.
