# Supplementary material for: Phenolic Contents and Antioxidant Properties of Bauhinia rufescens, Ocimum basilicum and Salvadora persica, Used as Medicinal Plants in Chad
Source: Molecules. 2024 Oct 2;29(19):4684. doi: 10.3390/molecules29194684 (PMC11478144; doi:10.3390/molecules29194684)
Supplement: Supplementary file 1 [file molecules-29-04684-s001.zip › molecules-3167896-supplementary.pdf]

Supplementary data

Phenolic Contents and Antioxidant Properties of *Bauhinia rufescens*, *Ocimum basilicum* and *Salvadora persica*, Used as Medicinal Plants in Chad

Abdel-razakh Hissein Hassan [1,2,3], **Gaymary George Bakari** [2,4], **Jin-Soo Park** [3,5,*], **Cheol-Ho Pan** [3,5] **and Abubakar Shaaban Hoza** [1]

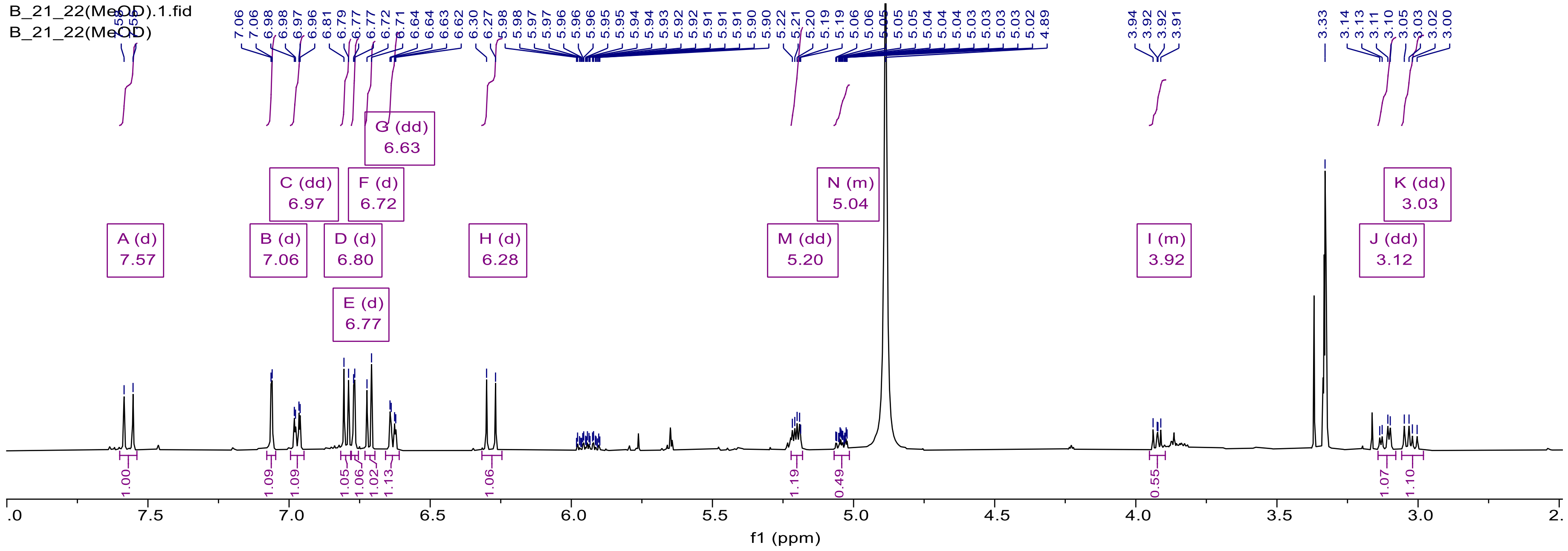

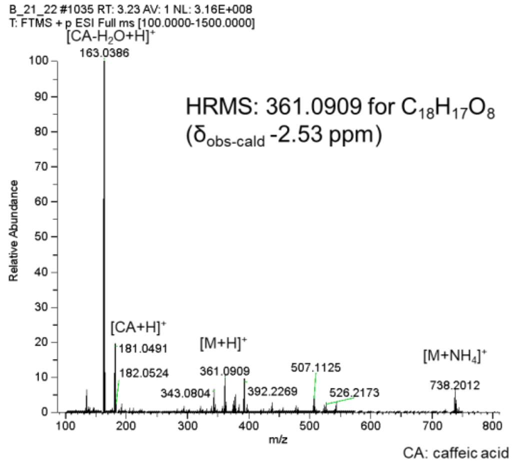

Figure S1. ^{1}H NMR spectrum and high-resolution mass spectrum of rosmarinic acid isolated from *O. bacilicum.*

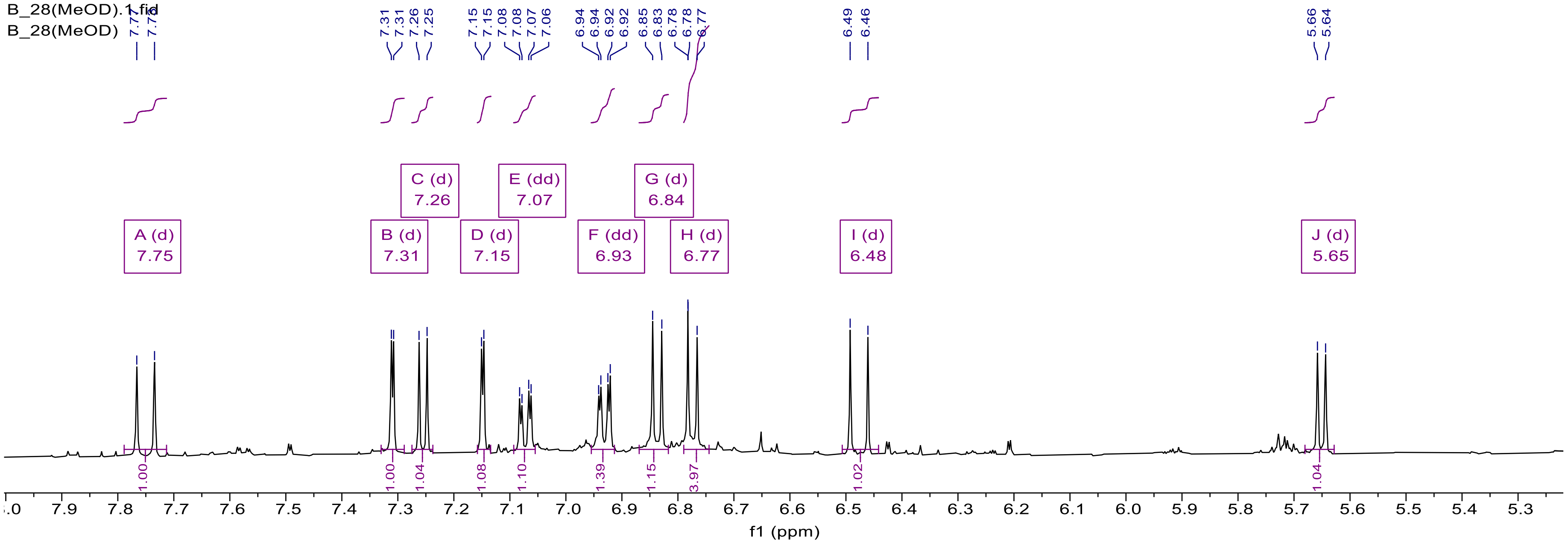

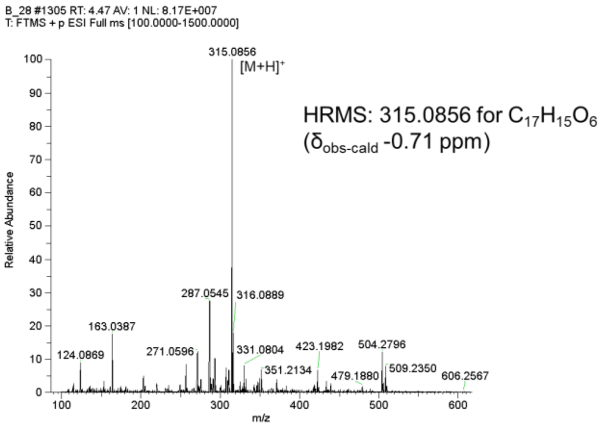

Figure S2. ^{1}H NMR spectrum and high-resolution mass spectrum of neopetoidin B isolated from *O. bacilicum*.

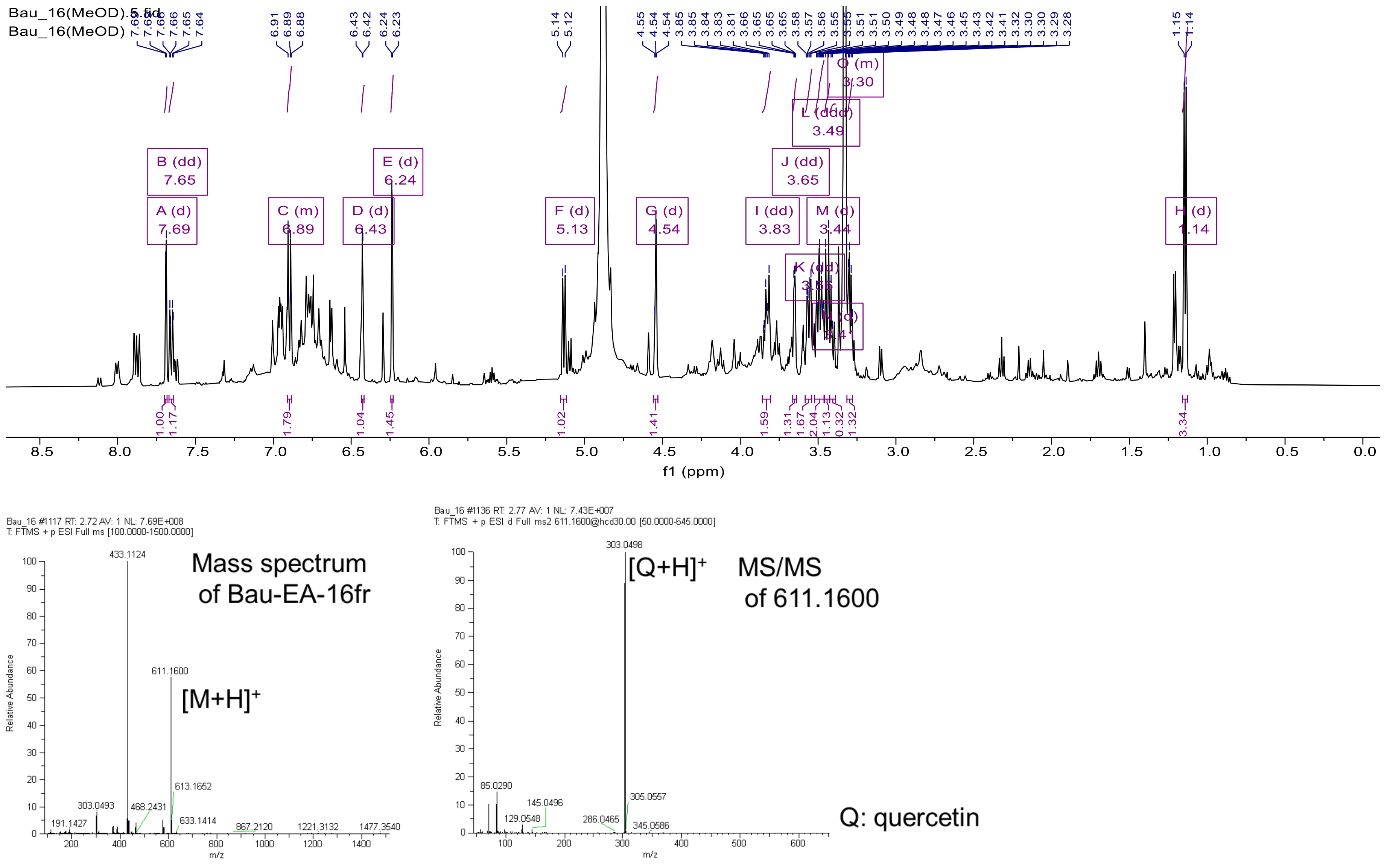

Figure S3. ¹H NMR spectrum and high-resolution mass spectrum of rutin isolated from *B. rufescens*.

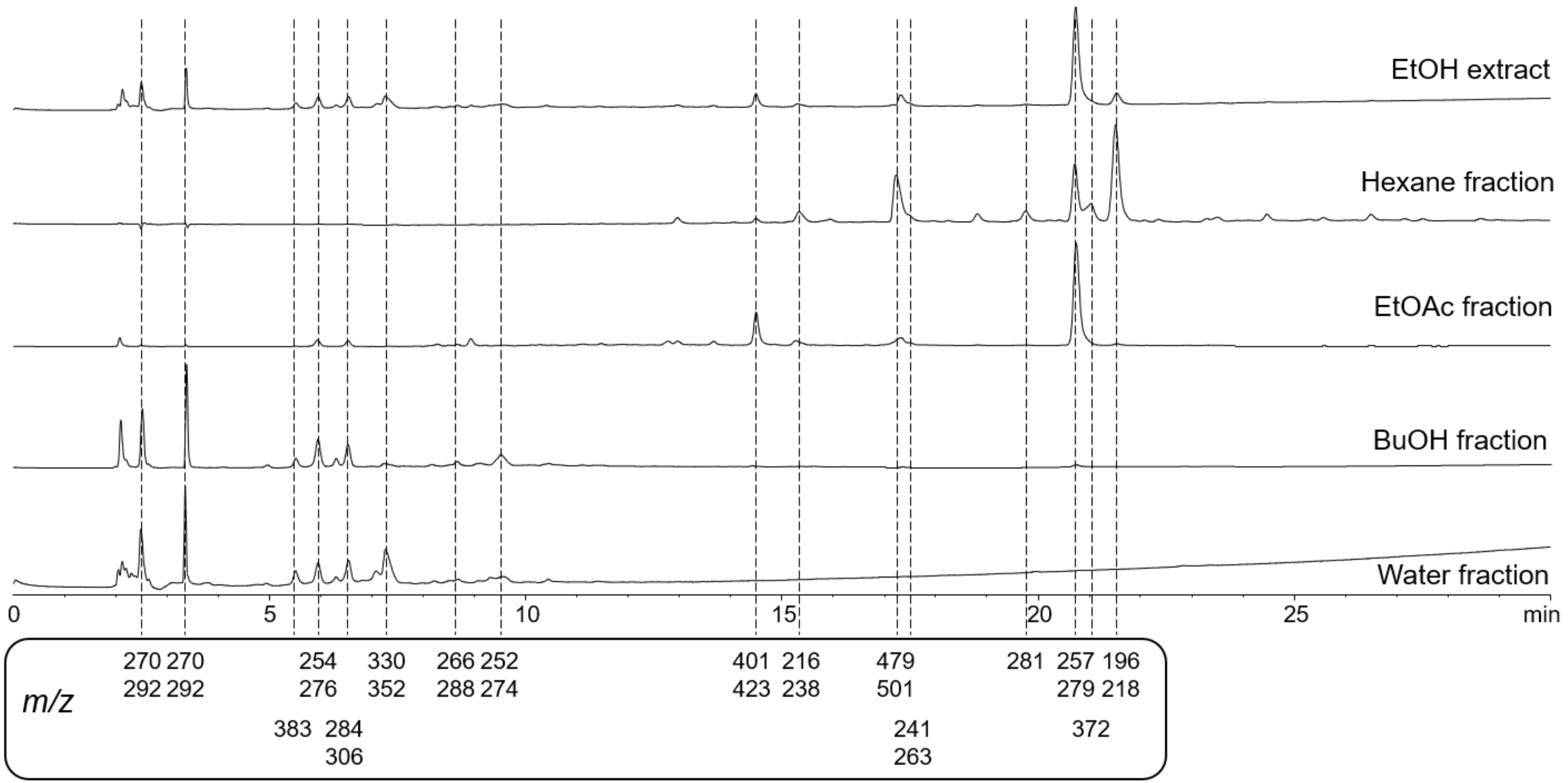

Figure S4. LC-MS data of extract and solvent fractions derived from *S. persica*. The molecular ions of mass spectrum corresponding to each peak are shown below the dotted line.

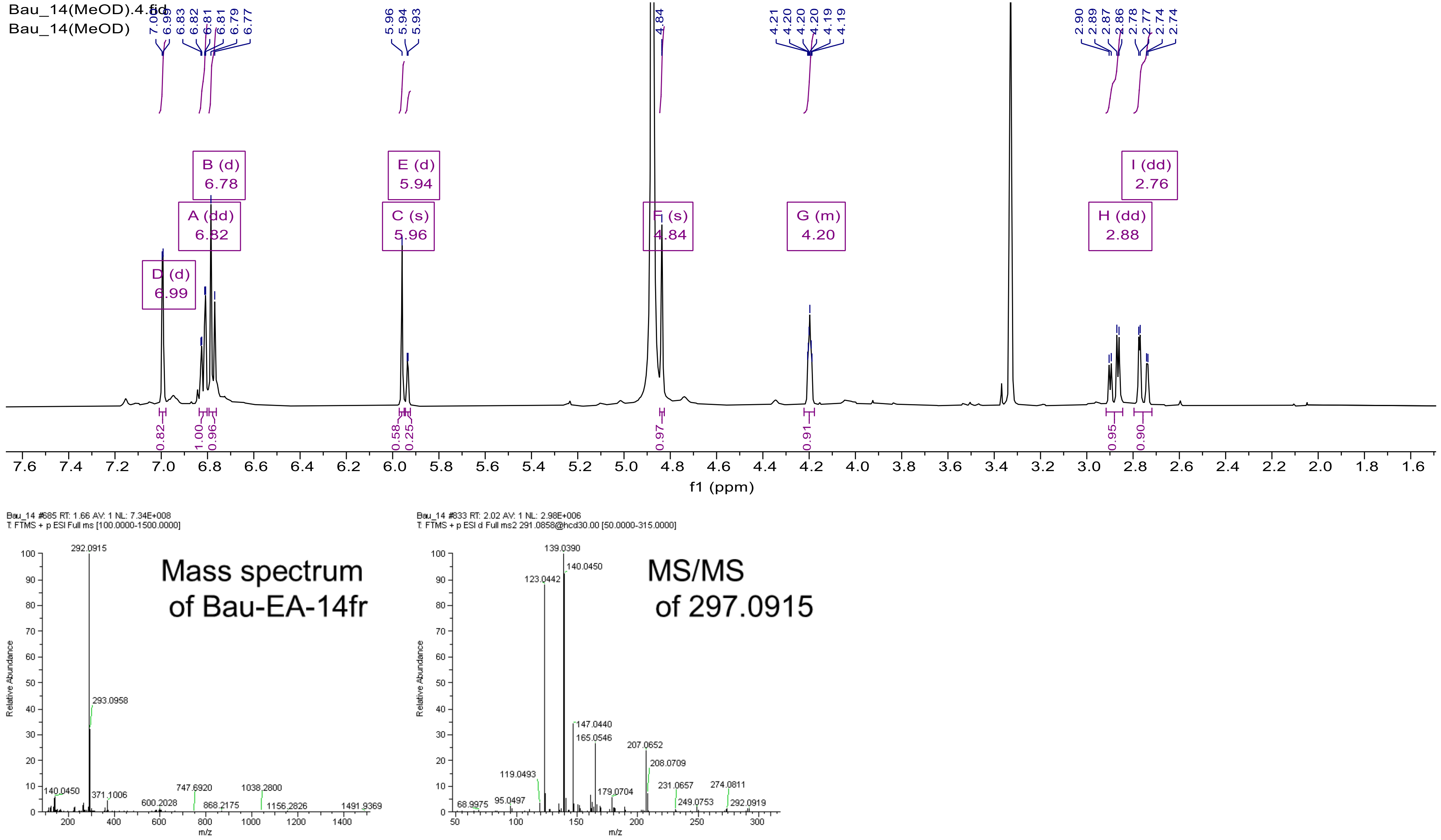

Figure S5. ^{1}H NMR spectrum and high-resolution mass spectrum of epicatechin isolated from *B. rufescens*.

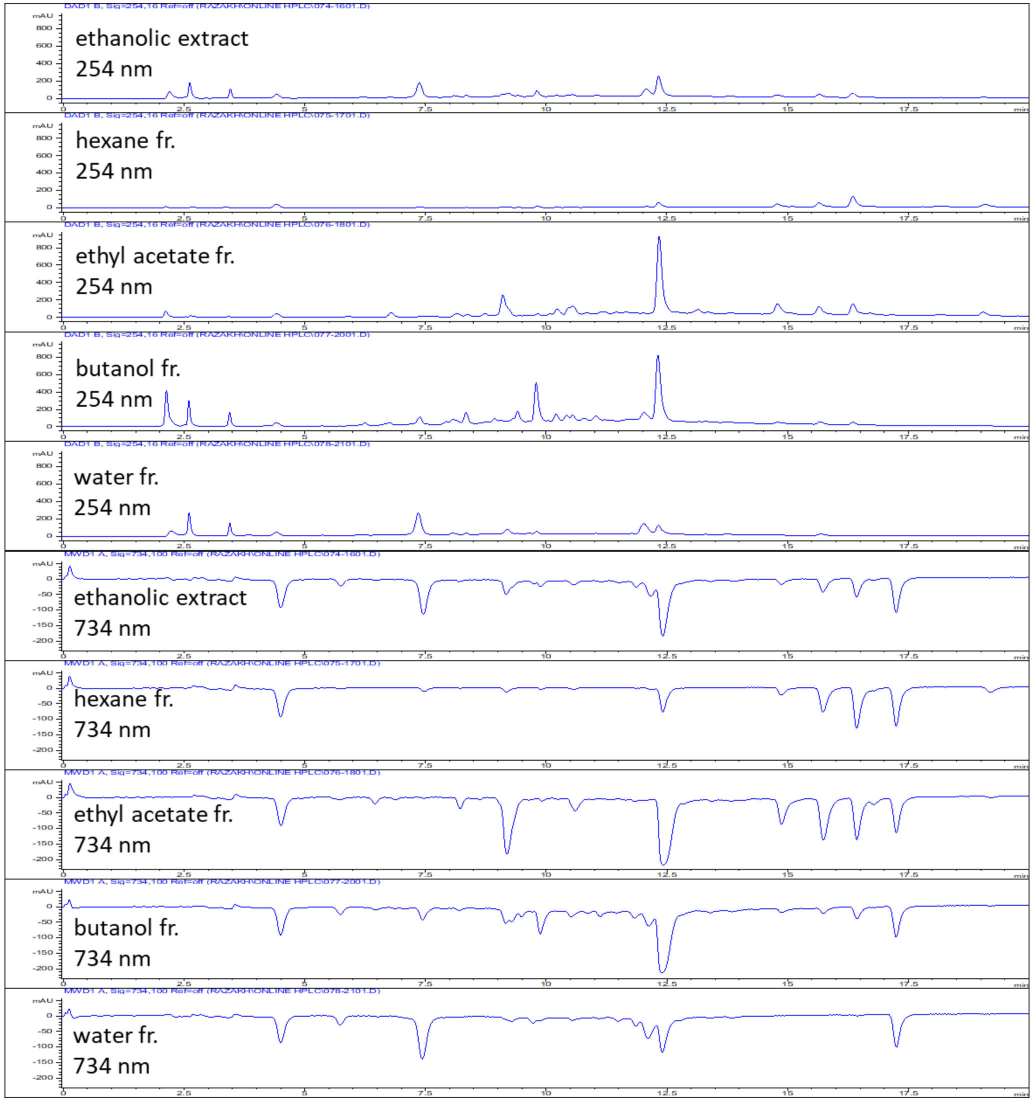

Figure S6. Chromatograms (254 nm) and radical scavenging activities (734 nm) of the ethanolic extracts and fractions of *O. basilicum* from online HPLC-ABTS system.

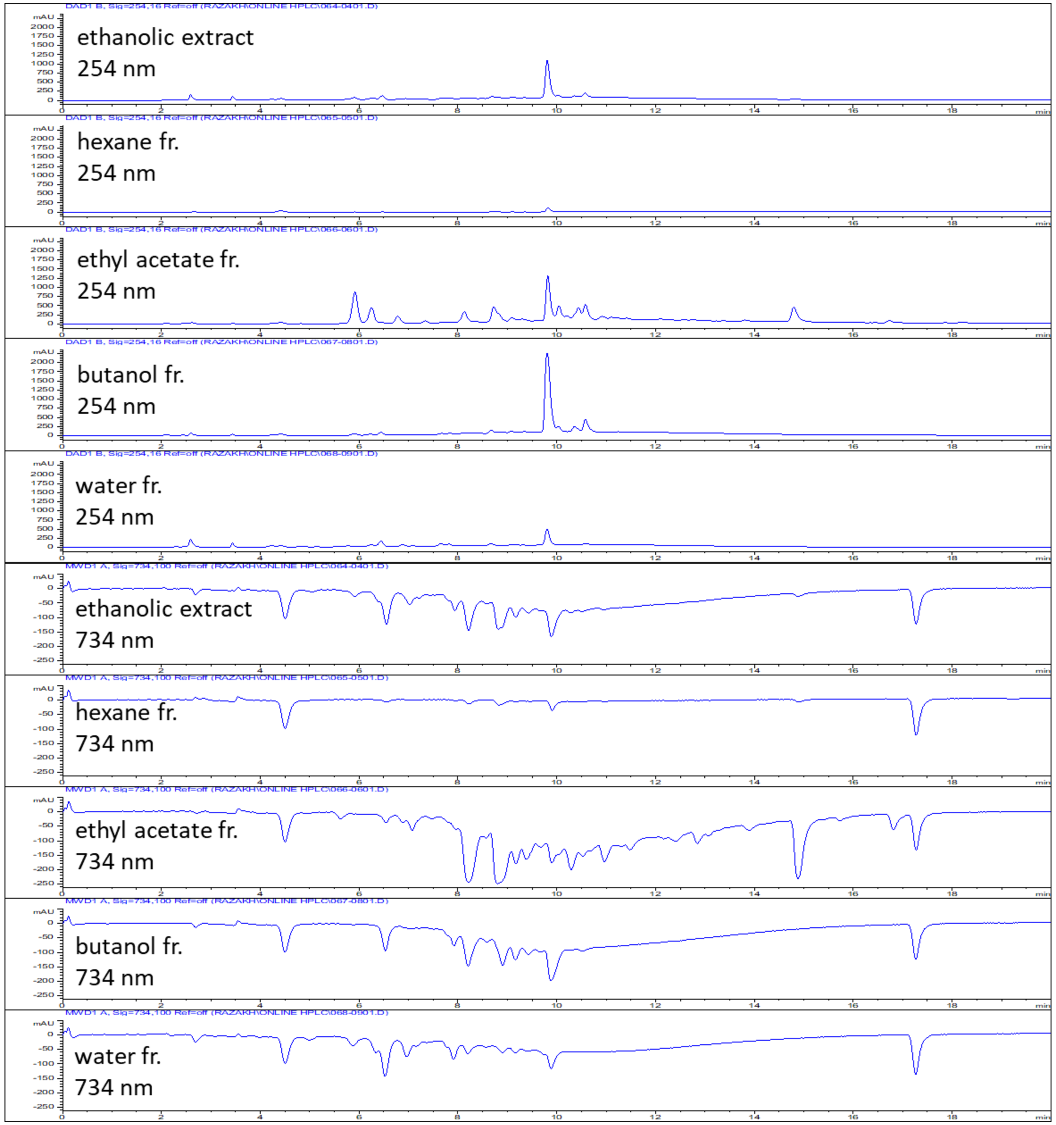

Figure S7. Chromatograms (254 nm) and radical scavenging activities (734 nm) of the ethanolic extracts and fractions of *B. rufescens* from online HPLC-ABTS system.

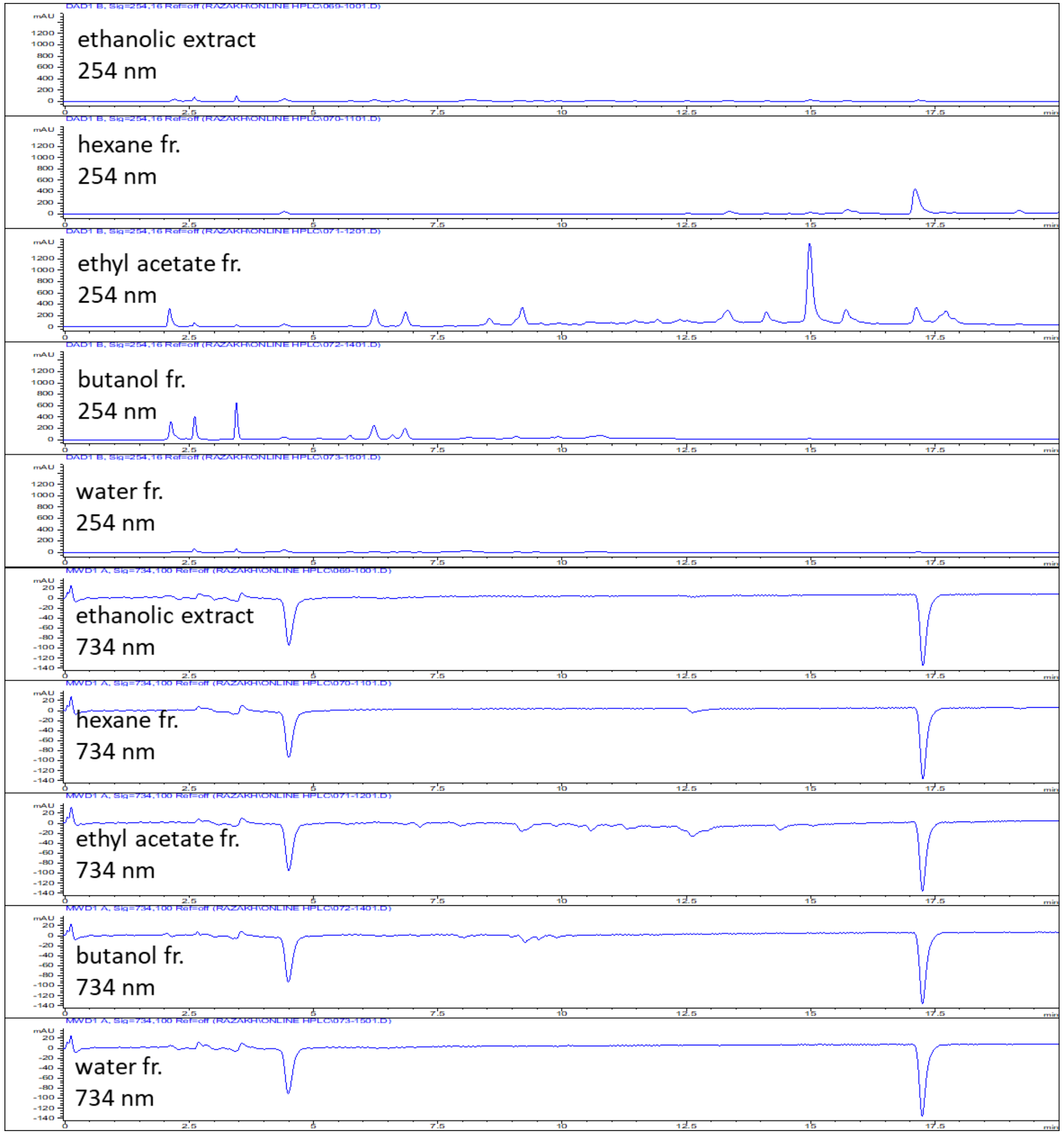

Figure S8. Chromatograms (254 nm) and radical scavenging activities (734 nm) of the ethanolic extracts and fractions of *S. persica* from online HPLC-ABTS system.
